# Nature–culture relationship process—toward constellar relationality

**DOI:** 10.3389/fsoc.2025.1441600

**Published:** 2025-06-10

**Authors:** Maya Aguiluz-Ibargüen

**Affiliations:** Centro de Investigaciones Interdisciplinarias en Ciencias y Humanidades (CEIICH). Universidad Nacional Autónoma de México (UNAM), Mexico City, Mexico

**Keywords:** culture-nature divide, culture-nature relations, anthropocene age, constellar relationality, worlding

## Abstract

In this article, I analyze the semantic genesis of the mutations experienced by the nature–culture binomial, which, in some cases, tends to be a continuum and in others, tends to the dualization of both domains. I begin with a brief analysis of the transcendence of the classical reflection on Nature from the phenomenology of Maurice Merleau-Ponty to stop my attention on the marked nature–culture duality in the no less classical formulations from specific works of Sigmund Freud and Claude Lévi-Strauss, even when this dualism is enriched by the conceptual variation in the work of each one. In the second part, I analyze the condition of possibility on which the above argumentation rests on the basis of a preparatory narrative that proceeds to objectify “nature” in the new modern cosmology that emerges in the seventeenth century with the stamp of Galileo and Descartes. In the third part, I analyze a whole set of sociological evidence that questions the plausibility of this separating duality between nature and culture. In the fourth part, I analyze the return of the nature–culture continuum that emerges at the hands of a new cosmology in late modernity that receives the stamp of James Lovelock and Bruno Latour.

## 1 Introduction

The separation between nature and culture has been constant in the Western worldview, at least since the mechanistic revolution of the seventeenth century, as represented fundamentally by Galileo and Descartes. Nature was de-daimonized and constituted as an external observable object of scientific experimentation and economic exploitation through the device of classification and entitative ordering, that is, culture. Freud (1981) and Lévi-Strauss (1981), each of them with their own methodological emphasis in very specific works, have confirmed this assumption and have established a sharp separation between the two spheres, situating the former as the static part as opposed to the latter, which has been considered as the dynamic part. The immanentist-materialist objectualization of nature, which reached its maximum expression in the mechanistic Revolution of the seventeenth century, consummated a great process of “disenchantment of the world,” as Max Weber had already seen, according to which the “Physis” became inert, passive, and mechanical matter.

However, the existence and persistence of a whole series of original ontologies—animism, naturalism, totemism, and analogism—embedded in the societies of the original peoples has allowed us to establish a “simultaneity of the non-simultaneous,” that is, we live in all these dimensions of time at the same time; however, there is no relationship of one-to-one simultaneity between the concurrent moments of events in each of these dimensions. This “simultaneity of the non-simultaneous” encompasses primordial ontologies (animist, naturalist, totemist, and analogist) that coexist in conflict with modern ontology, where the nature–culture continuum has been transformed into a duality by the cognitive hegemonization of the cultural over nature. As he added, this clash of cultural ontologies shows that we cannot define “nature” without simultaneously defining “culture” at the same time. As Latour highlights, we do not find ourselves before domains, but before one and the same concept that is divided into two parts that are linked. There is no other nature than this definition of culture and no other culture than this definition of nature. They were born together, as inseparable as Siamese twins who caress each other or have a fistfight while sharing the same trunk. Latour, in one of his latest works—which I analyze in detail in this paper—has shown how a re-enchantment, a re-animation, of Gaia (the name given to the Blue Planet, the Earth) has taken place. The telluric forces of nature manifest themselves as sociohistorical forces.

## 2 A deceptive duality in Freud and Lévi-Strauss: nature–culture

The constitution of a nature–culture has indeed begun! Not only do theoretical debates increasingly rest on the urgency of suspending a distinction between the domains of nature and culture, but it is true that this dualism stood out throughout the last century, denoting separate dimensions of human practice, tending to “construct” a nature willing to take on humanizing forms and characteristics, as a consequence of the long formation of an imaginary or assigned to nature a homogeneous entity. This framework, which undoubtedly prevailed, justifying transformations that undermined forms of life and their natures and habitats, showed its exhaustion, unfortunately, with the advance of the darker side of major changes inspired by the centrality of human anthropocentrism, as it was forged throughout the eighteenth century. The dualization between nature and culture, which will form the basis of some and specific works of both Freud and Lévi-Strauss -both children of their time, let us not forget that they wrote *Civilization and its Discontents* (1981 [1930]) and *The Elementary Structures of Kinship* (1981 [1949]) around the middle of the twentieth century respectively-, is inscribed in the important changes that take place from the seventeenth century onward within modern ontology (Mumford, 1971, p. 60ff; Koyré, 1979, pp. 87–107; Mayr, 1986, pp. 54–102). The technical dimension of the objectification of the real is, of course, essential in the mechanistic revolution of the seventeenth century, which represents the world in the image of a machine whose gears can be disassembled by scientists, and no longer as a totality composed of humans and non-humans and endowed with an intrinsic significance by divine creation. Lenoble (1969, p. 312), a historian of science who died in 1959, assigns a date to this rupture: 1632, the year of the publication of Galileo’s *Dialog on the two highest systems of the Ptolemaic and Copernican world*, where modern physics emerges, in the arsenal of Venice, from a discussion between engineers trained in the mechanical arts, a thousand leagues away from the *disputatio* of philosophers on the nature of being and the essence of things.

According to Maurice Merleau-Ponty, in his last courses at the Collège de France between 1956 and 1960 (Merleau-Ponty, 1994), studying nature motivated approaches about subjective co-constitution. Enigmatic phrases such as “it was not the scientific discoveries that provoked the change of the idea of nature, but it was the change of the idea of nature that allowed those discoveries” (1994, p. 25) give an account of a human understanding transformed from the incarnated experience.

As part of a working hypothesis pursued in this article, in Merleau-Ponty’s last reflection, there is a removal of the anthropocentric substrate of social theory as a consequence of the recognition of a surrounding world (to use a translation of the term Umwelt), distinct from the human Umwelt, but equally available to be read, captured, perceived, lived, and acted upon from the relational bodies in the thresholds of a nature–culture continuum.

It is not possible to detail how the above suspends some of the most recurrent anthropocultural conceptions. By way of example, I am thinking of Sigmund Freud when he places the origin of human culture where a family is founded for the first time (Freud, 1981, pp. 303ff), the first unit formed by isolated individuals. The family arises at the same time from a double necessity. Since the impulse toward genital sexual satisfaction was in the human being (by then an emphatically masculine being), unlike what happens in animals, it was permanent since the male experienced the need to keep his sexual object close to himself. On the other hand, the need for defense against an external “Nature” (with a capital letter) that is hostile to the human being (scarcity and difficulty in obtaining food, cold, heat, wild animals, etc.) leads the latter to appreciate the value of his fellow men as collaborators in the work, in the task of dominating that hostile “Nature” and putting it at his own service. *Eros* and *Ananké* appear in Freud as founding forces of culture (although we will not analyze now what have been the cultural routes of the death drive). Following the extension of this original family unit, Freud proposed the creation of a fraternal clan after the murder of the chief of the primitive horde, the father, perpetrated by his sons (Freud builds this idea on the proposals of Darwin, Atkinson, and Robertson-Smith). This first extension was followed by others, more and more extensive: tribes, cities, and nations. The development of culture thus brings with it a growing expansion of the human community and, due to the empowerment of labor, also a growing domination of the human being, let us say, man, over the externality represented by “Nature.”

In accordance with some ideas of Lévi-Strauss, the mediation between nature and culture takes place in and through the human mind, which always functions in the same quasi-linguistic structural mode, opposing matter or natural chaos as a cultural form or configuration. This is exemplified in the prohibition of incest (Lévi-Strauss, 1981, pp. 41–2) which constitutes the passage from nature to culture, a passage that is realized in and by a process of socialization. That is to say, it is in the exogamic deployment, in the natural necessity of sociocultural exchange—of women, goods, and messages—that Lévi-Strauss’ teacher Marcel Mauss had already noticed in his masterly *Essai sur le don* of 1924 (Mauss, 1971, pp. 155–267)—expressed as a form of social communication where our “expulsion” from nature and our entrance into culture would become evident.

Without denying the importance of both the family and the incest taboo in modern ontology, however, we will now analyze a whole series of arguments that question the universality of the separation between nature and culture, i.e., the opposition between nature and culture does not really have the universality that is attributed to it. The most powerful criticism comes from the analysis of the following four ontologies: animist, naturalist, totemist, and analogist. Among existents, the hegemonic role that modern ontology has attributed to the *anthropos* among existents is clear, but all that collectivity of existents linked to it—the non-humans, animals, plants, and later machines—have been relegated in modern ontology to a function of separated environment, and all of them now viewed as agencies have questioned the monopoly of “humanity” attributed exclusively to the *anthropos.*

## 3 The preparatory narrative of the divide: the objectualization of “nature” in the new modern cosmology of the seventeenth century

The social and semantic genesis of the concept of “nature” can be enormously revealing. *Physis,* that is the name we have inherited from the pre-Socratic Greeks to designate the phenomenon par excellence, so precise and determined, so ostensible, which is the *sensible phenomenon*. It has been translated into Latin and from Latin into all European languages, using the term “nature.” Both etymologically derive from roots, meaning to *be born and to grow*, to *sprout*, to *make grow*, especially of plants, that is, the whole ontogenetic process and ontogenesis of living organisms starting from the seed, and the intimate properties related to that process. The root of “*Physis*” is “bhu,” from the Sanskrit “*bhuti*” (the “born,” the “grown”), to come into being, to come into existence, to be born, and the root of “natura” derives from the Indo-European “*jan*, *jen,*” which likewise means to be born or to beget (Benveniste, 1969).

However, I prefer to use the term “*Physis,*” because for us, it preserves, more intact and purer, its primitive dedication to the world of the senses. The term “*Physis*” remains for us, still, much closer to the original intuition that created it. Homer uses it once to precisely designate the external appearance of the magical herb ‘molu.’ Through the mouth of Ulysses in *The Odyssey,* he says, “Having spoken thus (the God), Argeiphontes (Hermes) plucked from the earth a magic herb and showed its appearance. Its root is black, and its flower is white, like a milt. The gods call it “*molu,*” and it is very difficult for a mortal to pull up, but the gods can do anything” (Homero, 1981, X, pp. 302–5). It can be said that this term “*Physis*” still evokes the Homeric vision, primitive and naive, and uncontaminated, and it even preserves some of the *recent freshness of the being that has just been born.* This is capital here because “*Physis*” is the name of a phenomenon, of a phenomenal being, and the phenomenon or phenomenal being is precisely a being that *always exists in a state of nascence*. The phenomenon does not grow old; it does not even become old. It is born at all times, better continuously, as a manant source by virtue of its essential temporal unity. It is present in hatching, the auroral sun, and the sunset sun. *Physis*” alludes to the world of external phenomena, and from this sense would have come to designate the idea of *substratum*, which is at the base of all phenomena and to which it is intimately linked (Mansion, 2000; Hardy, 2010). *“Physis”* would be *matter, hyle,* yes, but a matter that does not coincide with our modern conception of matter as inert, passive, and mechanical, since it has, or is, at the same time, *zoé*, a “*continuum* of living fluid” (Cornford, 1984, p. 110), eternal and infinite life, from which comes and in which an individualized form of life (*bios*) is inserted. Matter is no more understood in a hylomorphic manner. “That nature (…) is already from the beginning a metaphysical entity; not only a natural element, but an element invested with life and supernatural powers, *a substance that is also soul and God.* It is, then, that same living material from which *daimons,* gods, and souls were gradually taking shape” (Cornford, 1984, pp. 147, 155).

From the seventeenth century onward, nature became a scientific phenomenon or “*episteme.*” (And at the same time, nature began to admit methaphors) The term “science” corresponds to the Greek term “*episteme*” which is opposed to “*doxa*” (opinion), and means the noblest knowledge of man, that is, all rigorous, methodical, certain, and demonstrable knowledge. This Greek scheme, refined by Plato (especially in The Republic, VI 509 d–VII 521 b; Platón, 1981) and by Aristotle (particularly in Second Analytic II, 156, and Metaphysics VI; Aristóteles, 1982), and whose details we will not go into now, endured well the development of human thought until the Modern Age. However, from the seventeenth century onward, a new type of noble knowledge, with its own rigor, method, certainty, and demonstrability, developed vigorously. Since then, this specific knowledge has increasingly appropriated the use of the term “science,” restricting it to the point of making it almost synonymous with “exact science” or “science of nature” (Stegmüller, 1979, 1983).

Before the Western Enlightenment and secularization, the world was “a great enchanted garden,” in Max Weber’s terms (Weber, 1946, pp. 129–156). In the “enchanted” world, faith was not opposed to knowledge, nor was myth against reason. The realms of spirit and matter are porous and are not easily distinguished from each other. Then came the dawn of modern science, which turned the world into an area of investigation. Nature ceased to be a source of wonder and became a force to be mastered, a system to be deciphered. At its root, “disenchantment” describes the fact that everything in modern life, from our minds to the rotation of the planets, can be reduced to the causal mechanism of physical laws (O'Gieblyn, 2021, pp. 6–7; Mayr, 1986, pp. 54–102). In place of the *pneuma*, the spiritual force that once infused and unified all living things, we are now left with an empty shell of gears and levers or, as Weber put it, “the mechanism of a world stripped of gods.” Using Spinoza’s terminology in his *Ethics*, we can notice the semantic change that goes from premodern *natura naturans*, from that dynamogenic potentiality with infinite capacity for engendering (*mater omni parens*), to protomodern *natura naturata*, to pure inert, reanimate, reified matter (Spinoza, 1977, Part One, Proposition XXIX, Scholium).

It has been a long and entrevered course since human understanding first put forth the objects that exist before and without us. Certainly, in its frame, the world was conceived as the totality of all representable objects. That idea got matter when Kant’s *a priori* categories of knowledge because, for him, there were objects out of reach. Later, at the turn of the twentieth century, the phenomenological approach opened the object, the thing, which seems to have become “simultaneously closer” (by intuitive perception) and further from the “perceptual mastery of the subject” (Hudek, 2014, p. 15). The subject confronted the question of representation.

The development of science shares with metaphysics the fiction of considering the world in its totality as something observed from a completely *transcendent* point of view. This point of view of a divine observer must allow the human spirit—regardless of its organic incorporation and symbolic structuring, its social location, and the historical situation of its “being-in-the-world”—a “look from nowhere,” an abstract, theoretical, aseptic, third-person look. The nominalism of William of Occam considers universals as concepts alien to reality, mere names, and simple ways of referring to things. Before an ontology of more ordered contingent events emerges, the subjective cognizing spirit must forge concepts for itself to explain the facts of nature as well as to interpret the meanings within the social world. The dualism between the individual and the world and the dualism of nature and culture (Latour, 2010) become a key concept in cosmology in which things subject to laws and the thought that organizes them into signifying sets, the body turned into a mechanism and the soul that governs it according to the *daimon* of each individual, are face to face. If Weber viewed a disenchantment in a new role of rationality that dominated different spheres of life, somehow there was a process of *de-dæmonization—the loss of the daimon voice—*consolidating a process of *objectification at all* based on a knowledge of nature as the other that describes the transition from theology to philosophy and from philosophy to science.

In the 21st century, complex systems between (and within) countries, companies, industries, and society as a whole have changed. There is an *increase in systemic complexity* (Luhmann 1980), a selective increase in the connectivity of different elements within systems and of the systems themselves among themselves. Not only do the “what” and “how” to do things change, but the “*who we are*” has really changed. The classical human subject (from Descartes onward) is de-subjectivized, de-centered, becoming, to a large extent, *another* element in the midst of the convergence of integrated systems in the acronym NBIC: nanotechnology (nano), biotechnology (bio), Big Data (Info), and Artificial Intelligence (cogno; NBIC; Roco and Bainbridge, 2003) that form “hybrid cognitive collectivities” (Donald, 1991, pp. 355–60) in dynamic association and interaction (Latour, 2005, pp. 1–21; Ikegami, 2011, pp. 1, 155–84). According to this, the science of the social is a *mapping of associations*. Here, the social does not mean one thing among others, like a black sheep among white sheep, but a *kind of connection* between things of which not all of them are social (Latour, 2005, p. 5). Haraway (2016) and García Selgas (1999) put us in front of the irrefutable fact of a progressive crumbling of certain epistemological boundaries: between the human and the animal, when humans carry in their own organism implants and transplants of animal parts; between the organism and the machine, when we carry in our own organism nanomachines that regulate functions of the organism; between the physical (the material, the hardware) and the non-physical (the formal, the software); and between the natural and the artificial. The so-called “surrounding world” (*Umwelt*) by biologist Jakob von Uexküll and of “hybridization” by Verbeek help to understand that there is no single operative force; instead, there is a diversificated and co-constituted world “where the object, whether thing, tool, commodity, thought, phenomenon or living creatura, has regained its ighst, freed form the subject’s determining mind, body and gaze” (Hudek, 2014, p. 16). Rather than an ontology of being, in classical terms, we should place ourselves in the perspective of *plural ontologies of existents*.

## 4 Socio-anthropological shifts question the separating division between nature and culture. The disenchantment of the “disenchantment of the world”

More than two million years ago, the hominid genus (Australopithecus) with at least seven different species began to walk upright but also climbed trees in southern Africa; after them, Homo erectus used fire; nevertheless, paleoanthropology has also paid attention to their vegetarian diet. Food-conditioned relations with other species and the capacity for mobility conditioned the management of space and distance from others. A first wave of physical and land expansion began through hunter-gatherer collectives (Ingold, 1996, pp. 117–155; Graeber and Wengrow, 2021) that represents a process of cultural diffusion that will take it on a long-lasting transcontinental journey through the Fertile Crescent, Asia, crossing on foot at that time the Bering Strait, which links Asia and America, descending the Canadian northwest coast and the North American Great Plains, crossing Central America, and extending throughout South America. In this journey through time and space, a great diversity of cultures has been generated, configuring ontologies, and classificatory schemes, as well as diverse ones. The metaphor of a permanent journey that begins in Africa and ends in an open destination is part of human existence. Becoming is geographical and historical; that is, it unfolds spatially and temporally in the sense of an open coevolution of contiguity and succession. Spatial contiguity allows for the *multiple and simultaneous unfolding* of becoming, which is not transferred from one state to the next through a chronological sequence of events but branches out and spreads *rhizomatically* in all directions. The birth and extension of universal religions will represent a second wave of land expansion (“territorialization”); this time no longer physical but ideational, those of the territorial discoveries and the opening of the great circumnavigations and trade routes (commanded by circumnavigators or Argonauts such as Columbus, Magellan, Elcano, Nunez de Balboa, Vasco Da Gama, and Captain Cook), which in time became colonial expeditions with more economic nature.

The notion of “simultaneity of the non-simultaneous” takes us back to the Christian regime of historicity. St. Augustine theorized it (without naming it) and placd it at the heart of universal history. The whole history of the two cities—that of God and that of the Earth—which has accompanied us ever since, relates their joint and at the same time distinct march, always crossed by the experience of the simultaneity of the non-simultaneous (Hartog, 2022, p. 229; Hartog, 2024). This experience has increased in modernity since the seventeenth century. The term ‘non-simultaneous’ means that qualitatively different stages of development appear ‘simultaneously’ within the same quantitatively measurable time (the time of the clock, abstract, universalized, with its time zones). The contact with native populations boosted the sensation of acceleration and modern progress, and at the same time, this gave rise to the unfolding of constructions about the self and the other, the representation of the self and the others.

The root of this contrast refers to the confrontation at the end of the fifteenth century between the European culture that interprets itself as the advanced world culture and the Mesoamerican and South American cultures, lowland and highland, of the so-called conquered new world, interpreted by the former as primitive and less developed (Koselleck, 1993, p. 290; Fabian, 1983) or the rural and indigenous people as anachronistic or pre-political subjetcs (Chakrabarty, 2000, p. 13; Bhattacharya, 2011). It is in this singular experience of time that different globally spatialized ontologies—animists, naturalists, totemists, and analogists—confront and clash with the hegemonic modern ontology and where the very modern separating duality between nature and culture loses its explanatory force. Philippe Descola puts it this way: “Contrary to modern dualism, which deploys a multiplicity of cultural differences against the background of an immutable nature, Amerindian thought considers that the totality of the cosmos is animated by the same diversified cultural regime, if not by heterogeneous natures, at least by different ways of apprehending one another. The common referent of the entities that inhabit the world is not, therefore, man as a species, but *humanity as a condition*” (Descola, 2012, p. 36, my italics).

It was Merleau-Ponty who specifically introduced the massive and sensitive gesture of the lived body in the formation of space and of the world, a point of strong consequences for, just within anthropology, situating the singular nexus of the human being with a creative growth within the unfolding field of relations (“the human being as a singular nexus of creative growth within a continually unfolding field of relations”; Ingold, 2011, p. xii). A shared conclusion at this point in the development of phenomenology is that, for this moment, common sayings concerning “one’s own body,” in phrases such as “a/my body in space or in place,” gave way to a bodily instance that does not pass either as isolated materiality or as an idea or an expanded concept, but as a means of embodiment, and the “existing” body (the living) constitutes a milieu where flesh and existence compose each other and provide reciprocal depth and dimension.

The singularity of the human body is understood by its spatiotemporal contraction, situated in the midst of other non-human animal existences. There is, in the background, a different notion of modern metaphysics in which humanizing and humanizing action changes human places, other living beings, other natures, and their worlds. However, in the face of this, a multiple composition of being-in-the-world also opened up in which Merleau-Ponty admits the individuation of all the “existents” mentioned in a couple of lines above. In an exercise of reflexive regression to signify life from experience (a word rooted in the sense of “*peira*” which means attempt, test), New Zealand anthropologist Jackson (1996) observes that the suffix “ex” (out of), accompanied by “peira,” which in turn, preserves the root “per,” that in the Germanic “*fahar*” (To travel), serves to recognize in experience a category of the present and the future, both wrapped toward a meaning not yet specified (Irving, 2017, pp. 71–2), “to the most original layers of existence, where indistinction is constitutive of all distinction: [that goes] from consciousness to the body, from the body to intercorporeality, from intercorporeality to animality, from animality to an ontology of the sensible” (Cladavakis, 2016, p. 89) to be able to signify life from the experience of the sensory thresholds that unite and separate the cohabitation of the human body with other bodies.

This Merleaupontian horizon summarizes a key in the human-animal interspecies formation that vindicates the body of perception and movement within the notions of being and social subject, but now girded by its constitutive relations with environments and landscapes (surroundings), anthropological themes newly focused after 1980 until the formation of Tim Ingold’s concept of “local landscapes” in the process of dwelling and residing (Ingold, 2000, p. 186).

We see the emergence of an active “body-existence” (or in an anthropomorphic approach, a subject-body), with mobility and displacement, which affects and is affected, in a process in which neither the representational determinations of consciousness nor representation regulated by the framework of culture interfere beforehand, nor with a corporeality resulting from social inscriptions. In a central shift in sociological frameworks, we will see terms such as practice, habitus, and imitation, among others, being re-signified, naming now in another way these existences between bodies, sociality, and creativity of social praxis under the defining scopes of (1) individuation, understood as an unfinished process of constant relation with the environment; affect; and the experiential and sensitive dimension.

It is worth highlighting in the above, at least, a double helix of the views here only cited from the Merleupontian notes and from a short line of authors within phenomenological anthropology who admit a bodily dynamism in the fading of the culture/nature, human/non-human dualisms, and not least a “biology’s gift”: “an inherent dynamism of the body, a biological productivity [that also] fades the mind–body distinction.” (Papoulias and Callard, 2010, p. 34).

In this context, nature is not a transcendent instance or object to be socialized but the subject of a social relationship: it is an extension of the world of the family home, and it is truly domestic even in its most inaccessible redoubts. According to primordial ontologies, most entities that populate the world are linked to one another in a vast *continuum* animated by unitary principles and governed by an identical regime of sociality. The category of “person” encompasses spirits, plants, and animals, all endowed with a soul. This cosmology does not discriminate between humans and non-humans; it merely introduces a scale of order according to the levels of exchange between existents where there is a great “elasticity of borders in the taxonomy of the living” (Descola, 2012, pp. 35, 64). The way in which the modern West represents nature is the least shared in the world; that is, we Westerners are rare, while others represent the generality. In his book *The WEIRDest People in the World* (2020), Henrich (2020) accumulates hundreds of pages of data to demonstrate how unusual Western, educated, industrialized, wealthy, and democratic values are. As an example of this nature–culture *continuum,* Franz Boas describes the invocation of a Kwakiutl salmon fisherman from the Canadian northwest coast: “Welcome, Swimmer (referring to the salmon)! I thank you that I am still alive in this season when you return to our good place; the reason you return is because we play with the rigging together, Swimmer. Now go back to your home and tell your friends that you had good luck coming and may they come with their health-bearing message, Swimmer; and also take my illness with you, friend, supernatural Swimmer!” (Boas, 1921, p. 1319). In the same vein, an Amerindian informant of Philippe Descola stated, “The woolly monkeys, the toucans, the howler monkeys—all those we kill for food—are people like us. The jaguar is also a person, but it is a solitary killer; it respects nothing. We, the `whole people, ‘must respect those we kill in the jungle because for us, they are like in-laws. They live with their own kin; they do not do things at random; they talk to each other; they listen to what we say; they marry accordingly. We also, in revenge, kill in-laws, but they are always relatives. Furthermore, they might also want to kill us. In the same way, we kill woolly monkeys for food, but they are still relatives” (Descola, 1996; Descola, 2012, pp. 26–7).

We cannot define “nature” without defining “culture” at the same time. As Bruno Latour highlights, “We are not dealing with *domains*, but with one and the same *concept* divided into two parts that are linked…” There is no other nature than *this* definition of culture, and there is no other culture than *this* definition of nature. They were born together, as inseparable as Siamese twins who caress each other or have a fist fight without ceasing to share the same trunk” (2017, 29). Being aware of the modern resemantization of the concept of “nature,” it is better to use the typographical convention “nature–culture” in order to avoid turning nature into universal evidence upon which the category of culture would stand out within a process of *cognitive hegemonization* (Zerubavel, 2018, p. 57) that accelerates from the seventeenth century onward, in the same way that the use of “he/she” allows one to avoid taking the masculine gender for a universal, since there is the belief that there is a purely “human” culture, in which the difference between man and woman is irrelevant, supported by the naive identification of “human” with “man” (Simmel, 1999, p. 177).

In 1974, Sherry Ortner posed a key question in the title of her well-known essay, “Is the feminine to the masculine what nature is to culture?” In her view, at the time, in almost all cultures, women were considered to be closer to nature, and men were generally seen to be more usually associated with culture. Thus, these spheres of life, nature, and culture were gendered differently. At the same time, Ortner accepted a soft Marxist view, according to which culture can be defined by its capacity to transform nature. This view, according to which culture is defined by its transformative activity, while nature is there as a given object to be transformed by culture, is no longer valid today. It constitutes, in Judith Butler’s view (Butler, 2024, p. 225), a well-meaning but counter-ecological view that denies dynamism, agency, and transformative processes to nature.

We must be very careful to avoid the epistemological trap of considering the subject who sees a historical oddity by considering that which he looks at—a still life—as something *natural* or evident. There is an operator, an operation of *social optics* that *divides* object and subject that is also evident in that common concept that distributes roles within the nature–culture pair, as in the woman/man categories we have mentioned. There is something like a “hidden manipulative architect” (Latour, 2017, p. 33) who distributes roles between the role of nature (for a subject) and that of consciousness (of this object). This architect is not the God-Providence of Christian cosmology, nor its secularized version deployed as dynamic agency in Descartes’ *res cogitans*, but a social construction operated within modern cosmology that generates that separating duality between nature–culture from the 17th that *objectifies* “nature” and *subjectifies* “culture” and that we will analyze in more detail below. The natural world is *something more* than a mere reservoir that can be exploited by human beings (Castoriadis, 1975).

In natural law, the adjective “natural” appears as a synonym of “moral,” “legal,” and “respectable.” However, a closer look at the modern epistemological ruptures of which Foucault speaks to us in *Words and Things* reveals that there is a *semantic shift*, taken for granted, that hides the background that beats in the certification of a product labeled as “natural,” which in reality conceals another way of being “artificial” because the “nature” from which this “natural” product comes has been discouraged, objectified, reified, and is no longer nature but “nature.” In these processes of semantic displacement, when we speak of a “naturalized” position, we are implying something that has been “essentialized” beyond the effect of semantic erosion that it produces on historical events. The logical positivist Wittgenstein of the *Tractatus Logico-Philosophicus* clarified: “The world is all that is the case; it is the totality of the facts; *what there is are the facts*” (1973, p. 15, my italics). The world of being that governs classical ontology is now manifested in that *descriptive* character of the “natural” facts of modern ontology, in the *de facto* truths (Arendt, 2017, pp. 36, 55) that *order* the world, in the sense of a classificatory scheme, but which also *prescriptively order* some forms of action (Latour, 2017, p. 49), becoming *de jure* truths.

This “simultaneity of the non-simultaneous” that we have described above encompasses primordial ontologies—animists, naturalists, totemists, and analogists (Descola, 2012; Bergua, 2022; González-Abrisketa and Carro-Ripalda, 2016)—that coexist in conflict with modern ontology, where the nature–culture *continuum* has been transformed into a duality. The disenchantment of the world implies the existence and persistence of multiple collectives and ontologies.

The transition from *Homo erectus* to *Homo sapiens* (approximately 250,000 years ago) marks a new evolutionary stage, represented by *mythical culture*, characterized by the emergence of the speech system as a new modeling of the universe of human existence, as well as by the emergence of metaphor and narrative. This transition from a purely mimetic form of culture to spoken language, to narrative, and to a fully developed *oral-mythic* culture is a revolutionary development that precipitates a transition in representation from slow-moving mimetic customs to *group narrative capacity*. This adaptation introduces a new layer of culture with the consequence that both human cognition and associated cultural forms become more complex and diversified. The public expression of this new narrative ability manifests itself in a liberated imagination that empowers human beings to rearrange more complex events in the imagination or even to invent fictitious events, as occurs in narrative and fantasy, in mythologies, thus allowing the emergence of limitless variations in how group reality might be constructed (Donald, 1991, pp. 201–69). Innovation would be substantiated not so much in the communication articulated through the mimesis of gestures and signs characteristic of *Homo erectus*, that is, in the imitative ability to performatively reenact events, but in the reciprocal use of *symbols* that mean the same thing to the members of the group. This represents the *proto-enchantment* of the world. That group narrative capacity configures “schemes of integration of experience that allow us to selectively structure the flow of perception and the relationship with others, by establishing similarities and differences between things on the basis of the identical resources that each one carries in himself: *a body and an intentionality*” (Descola, 2012, p. 345, my italics). We usually think that the mimetic phase of our development as human beings is overcome by the symbolic phase, where we construct images and symbolic representations of reality, and that this phase is overcome in the conceptual-theoretical phase, where abstract thought makes a tabula rasa of all the previous, but this does not happen; *a new stage supposes rather a reconfiguration of old and new possibilities, instead of an overcoming and disappearance of the previous stages* (as opposed to the teleologism of modern ontology).

## 5 The last return of the nature–culture continuum in a new emerging cosmology in contemporaneity

At the XXXIV International Geological Congress held during the summer of 2012 in Brisbane, Australia, it was determined to consider the Anthropocene as a *possible* geological *epoch for the time being*, placed at the same hierarchical level as the Pleistocene and the Holocene, but that *the latter had ende* (quoted from Latour, 2017, p. 132). However, at a meeting of the same body on 1 February 2024, echoed by *The New York Times* on 5 March 2024, “geologists believe (not without strong internal discussion) that it is not time to declare a human-created *epoch*.” That does not invalidate the consideration of the Anthropocene as an undeniable sociocultural *event event* (Chakrabarty, 2009, p. 220; Bonneuil, 2015, pp. 17–31), in which differentiated humanity must come (Chakravarty, 2015). Or otherwise, it is a concept indicative both of the human being as a geological agent and of the critical threshold at a planetary level, at which point we also ask ourselves who will really bear this agency of geological transformation from now on (Biset, 2022 pp. 46–7).

It is as if there is a built-in thermostat that puts the brakes on continued development at each stage of development, a rather harsh thermostat that works through war, famine, and pestilence but does not stop growth, only prevents it from exceeding certain limits. The world of science and capitalism has removed the global warming thermostat, so we keep getting hotter and hotter, and nothing turns off the heat because there is no working thermostat. The idea of unlimited growth has no thermostat and is ultimately self-destructive (Bellah, 2024, pp. 24, 28).

Apocalypse and history do not usually go hand in hand. In search of the end that advances, attentive to its date, the apocalyptic does not expect anything from the past and expects—or expects and fears at the same time—an end to the present, which offers no other outcome. The impasse is total, and the outcome must come from elsewhere. For the prophet Daniel, the only remedy for the abomination of Antiochus IV was the coming of the Lord. All millenarian movements have been based on the hope of a new heaven and earth. From the chronos time,^1^ a time of misery, nothing good can be expected except an end. The phenomenon of the “*Great Acceleration in the Anthropocene*” (Steffen, 2015) is made explicit in that the data show that the dominant feature of socioeconomic trends is such that the economic activity of the human enterprise continues to grow at a rapid pace. Only after the mid-twentieth century is there clear evidence of fundamental changes in the state and functioning of the Earth System that go beyond the Holocene range of variability and are driven by human activities, so Antropocene designs the anthropogeneic climate change with its biggest cause, but it is no naming a “moral responsibility” (Chakravarty, 2015). Liberalism and Marxism, both discourses in nineteenth century, have overvalued industrialization and the boundless progress understood as the increase in productive forces, so the two have underestimated the staggering acceleration in the movement of the carbon cycle and the geophysical violence of climate, and after fossil fuels (coal, oil and gas) as causes of violence against life. In this way, planet Earth was a modern ontology for natural, social, and philosophical sciences since they have considered a foundational division between entities being and non-being, biology and geology, biochemistry and geochemistry, life and non-life (Povinelli, 2014).

However, Bruno Latour has found a place for history by designing a new cosmology that we should not overlook. Ulrich Beck constructed a sociology of nature in his 1986 *Risk Society*, while Latour discusses the makings of a different cosmology that overcomes the limitations of the one coming from the seventeenth century. Our place in the Anthropocene may, in his view, allow humans to rediscover the thread of history, a sense of history that has been taken away from them by what they had hitherto considered a mere framework deprived of any responsiveness (Latour, 2017, p. 61). They had called this context “nature” and had made it the backdrop of their world, their world with its own time and history. But now scientists have become the historians of nature. *Geology, with its billions of years, past and future, is a sociohistorical science*. Geological forms manifest themselves as strata of time, in that “becoming-stone,” the very history of humans and non-humans, is manifested. We should not, Latour writes, see Gaia (the name of the planet Earth, which he borrows from an earlier work by James Lovelock (1979)—the creative source of this new cosmology—who in turn borrows it from Greek mythology) as a cybernetic machine controlled by feedback loops, but as a series of historical events, hence a historicizing force (Latour, 2017, pp. 90, pp. 135, and pp. 245).

From Galileo’s conception expressed in the transit from the closed world (on itself) to the infinite universe (expressing the change from the geocentric to the heliocentric model), *we return*, with Michel Serres, from the infinite universe to the Blue Planet that demands for climatic justice, in stark contrast to Elon Musk’s proposals to conquer other planets. Serres takes as his starting point an episode in the history of science in the form of a comic strip: after the Holy Inquisition forbade him to publicly teach anything related to the motion of the Earth, Galileo muttered, “*Eppur si muove”* (and yet it moves), which prompts Serres to exclaim before the successors of the priests and prophets who are today’s intellectuals and scientists, “*The Earth shakes*!…. *The Earth trembles*” (Serres, 1990, p. 136), for the Earth has become a fragile, active, limited, local, sensitive, trembling, and irritable envelope, as evidenced by the indelible marks of the Anthropocene (Beuret and Brown, 2017).

For Latour (2017, p. 106), Gaia represents “the religious without religion,” where agents (humans) and actants (plants, animals and machines) are not prematurely unified, there is no totality, there is no Engineer who controls the design of complexity in its totality, there is no Plan-Providence, nor a Superorganism that acts on all the rest, rather it is about obtaining effects of connection between different possibilities of being and acting, without the conception and organic dependence of a totality, living in the midst of multiple realities, but, within one world made of many worlds, which is a strong planetary utopia so close to the “a world where may other worlds fit in,” from de the Mexican Zapatist movement. Existing entities (human and non-human) create their environment and do not adapt. It is clear that each acting power modifies its neighbors (Latour, 2017, p. 5 and p. 117) to make its survival slightly less improbable.

*Kairos* time fundamentally differs from *chronos* time, which is our measurable and fluid time. The former is open to the instant, to the unexpected, but also to the opportunity to be seized, to the crucial opening, and to the decisive moment. By giving a name to *kairos*, we give it a status and recognize that human time, that is, the time of well-regulated action, is a mixture of *chronos* time and *kairos* time. After Thucydides and his analysis of the Peloponnesian Wars, especially after the irruption of the Bible and the New Testament, time *Krisis* as disruption and transgression makes its appearance. To live as a Jew and as a Christian is to live two times simultaneously, existing in the present of ordinary time while not existing in it (the “as… not”). Keeping one foot in the *chronos* time while the other already treads the apocalyptic present of the Christic *Kairos*. In between are the moments of *Krisis*: the exodus in Egypt, the Babylonian exile, the dominion under Rome, and the modern Great Revolutions. To become a Christian is to learn to live in two incommensurable temporalities: in the eternity of God, which by definition is off limits, unquestionable, and unrepresentable, and in ordinary *chronos* time. To forge a connection between the two, *Christians made Jesus the Messiah, that is, the mediator: the Kairos*. In Latour’s proposal, the *Anthropocene, a geohistorical macro-event, serves as a new Kairos time*, a new opportunity. The difficulty lies in the fact that Latour subordinates the “plurality of times” existing today—evidenced between multiple, antagonistic temporalities of the *chronos* world, something that is expressed in the different climatic futures (Ramos, 2018; Latour, 2017, pp. 25–27; and pp. 232–233)—to the temporality of the *Um-welt* that the Anthropocene represents, something that we could accept as *wishful thinking* but that does not function as a proven fact. *The “simultaneity of the non-simultaneous,” as temporal and spatial implosion, is still copresent among us*.

If we think of time as an “*extended environment*” (Fraser, 1999), let us consider an arrow drawn on a sheet of paper. In principle, such an image can represent a whole plethora of structures and processes created by humans in the form of material and immaterial culture. This is how the *nootemporal* environment manifests itself. If we let the head and tail of the arrow become ill, the image is now a visual metaphor for a *biotemporal* environment of animals, children, and man in certain collectives, of certain fantasies and dreams; i.e., the time of living organisms appears here. If both the head and tail of the arrow are absent, we only have a line, the image of an *eotemporal* environment where the time of massive aggregates of matter, the astronomical world of galaxies, the Newtonian world, and the world of the theory of relativity appear. In this world, coincidences are chance simultaneities as opposed to the created simultaneities of noetic time. In the image of the arrow disintegrated into small dots or fragments of wood, the temporal positions in the world of atomic particles could be known only in probabilistic-statistical terms, offering a *prototemporal* environment, that is, the first of the series of environments. In the image of the blank sheet, time would have disappeared in the manner of a black hole in which the temporal dimension does not exist as the theory of relativity has shown, configuring an *atemporal* environment similar to the pre-Socratic concept of chaos, a state of things that precedes the formation of the world. *These are incommensurable temporalities*. We live in all these dimensions of time at the same time; however, there is no relationship of one-to-one simultaneity between the concurrent moments of events in each dimension. Attempting to synchronize the non-temporal rhythms of the world with the biotemporal rhythms is a very respectable thing to do, but it is part of the realm of the subjunctive rather than of the actual existing reality.

## 6 Conclusion

After this voyage through space and time, the semantic genesis, the wanderings, of this binomial that we have called nature–culture reveal that until the beginning of the “disenchantment” of the world, the *continuum* between existents—human and non-human—predominates, something that is reflected in the analysis of the four primordial ontologies. However, from then on, with different emphases, a process begins that accelerates in the seventeenth century and is characterized by the progressive de-divinization of the physical world, accentuating correlatively a vigorous process of objectualization of “nature,” which ends with a separated culture–nature and a world shaped by modern anthropos. To review how much we need to pluralize the world from a nature with many worlds (as in Viveiros de Castro’s sense of multinaturalism), I have chosen to use the term “Anthropocene” to leave questions like, “What kinds of human disturbances can life on Earth bear?,” which modalities of landscapes—human and non-human—are confronting us? (Gan et al., 2017, G12 and G1).

Certainly, there are landscapes of ruins and devastation, and they are part of the “simultaneity of the non-simultaneous,” that is to say, in the Latourian cosmology, Gaia emerges with her own voice, with the name of an angry and angry goddess. She challenges us to assume that there is no road map, no plan, no established end, but creative worlding inhabited by a new relationality among ecologies of non-life, life, and afterlife.

## Footnotes

^1^A semantic shift between *Kronos* and *Chronos* has meant that *Chronos*, ordinary time, has long been seen as *Kronos*, the Greek devouring God or the scythe, represented as Saturn devouring his children or Father Time equipped with his scythe. *Kronos* would appear as a symbol, a metaphor of time that ends all.
